# B2M overexpression correlates with malignancy and immune signatures in human gliomas

**DOI:** 10.1038/s41598-021-84465-6

**Published:** 2021-03-03

**Authors:** Hao Zhang, Biqi Cui, Yulai Zhou, Xinxing Wang, Wantao Wu, Zeyu Wang, Ziyu Dai, Quan Cheng, Kui Yang

**Affiliations:** 1 Department of Neurosurgery, Xiangya Hospital, Central South University, Changsha, 410008 Hunan People’s Republic of China; 2 Department of Neurology, Xiangya Hospital, Central South University, Changsha, 410008 Hunan People’s Republic of China; 3 Department of Oncology, Xiangya Hospital, Central South University, Changsha, 410008 Hunan People’s Republic of China; 4 National Clinical Research Center for Geriatric Disorders, Xiangya Hospital, Central South University, Changsha, 410008 Hunan People’s Republic of China; 5 Department of Orthopedics, The Third Xiangya Hospital, Central South University, Changsha, 410008 Hunan People’s Republic of China; 6 Department of Clinical Pharmacology, Xiangya Hospital, Central South University, Changsha, 410008 Hunan People’s Republic of China

**Keywords:** Cancer microenvironment, CNS cancer, Tumour biomarkers

## Abstract

Because of the limited treatment strategy of gliomas, the key of diagnosis and treatment is finding new molecular biomarkers. Here, we explored the potential of β2-microglobulin (B2M) to serve as a hopeful candidate for immunotherapy or diagnostic biomarker in gliomas. The genomic profiles, clinical characteristics, and immune signatures were analyzed based on TCGA and CGGA databases. We carried out the whole statistical analyses using R project. High B2M expression correlated with worse prognosis. Somatic mutations of gliomas with high B2M expression are associated with PTEN deletion and EGFR amplification. Isocitrate dehydrogenase (IDH) mutations accounted for 82% in gliomas with low B2M expression. In addition, B2M positively correlated with ESTIMATE scores, interacted with infiltrating immune and stromal cell types. B2M also suppressed anti-tumor immunity through immune related processes. Meanwhile, B2M was associated with immune checkpoint molecules and inflammatory activities. Finally, functional annotation of the identified B2M related genes verified that B2M was a potential candidate for immunotherapy. We confirmed that B2M played a critical role in tumor progression, patient prognosis and immunotherapy of gliomas.

## Introduction

Gliomas, originating from the supporting glial cells of the central nervous system (CNS), are the most common malignant tumors of CNS in adults^1,2^. Based on the World Health Organization (WHO) classification, gliomas are classified into grade I (lowest level) to grade IV (highest level) according to histopathological features. Low-grade gliomas (LGGs) refer to grade II and grade III tumors characterized by cytological atypia, including astrocytomas, oligo-astrocytomas or mixed gliomas, and oligodendrogliomas^3^. Invasive LGG is a slow-growing brain tumor that usually occurs in young or middle-aged adults. LGG continues to grow and usually turns into a higher grade of malignant tumor, eventually leading to progressive disability and premature death^4^. At present, the comprehensive prognosis prediction combined with tumor histology, tumor markers and tumor genotype has been updated frequently for the clinical management of LGG patients.

β2-microglobulin (B2M), a non-glycosylated protein, has a molecular mass of 11,800 Da. All nucleated cells could synthesize B2M which forms an immutable major histocompatibility complex (MHC) class I antigen small light chain subunit through non-covalent binding on the cell surface. MHC-I has been demonstrated to be an inhibitory receptor for NK cells, and loss of cell surface MHC-I due to B2M loss by cancer cells would render cancer cells more vulnerable to NK cells^5,6^. Correspondingly, B2M-deficient tumor metastasis was found to be significantly targeted by natural killer (NK) cells^7^. Based on single cell sequencing analysis of follicular lymphoma, B2M coexpressed with immune checkpoint molecules in regulatory T (Treg) cells^8^. The most characteristic role of B2M is interacting with the tertiary structure of MHC-I α-chain, thereby presenting antigenic peptides to cytotoxic T lymphocytes (CTLs). During the process of recognizing the foreign peptide antigen on the cell surface, T cells will actively bind and dissolve the cancer cells presented by the antigen. In mice with B2M deficiency, defective antibody responses are observed since the sensitivity of NK cells to MHC-I heavy chain mediated inhibition increased and IgG catabolism increased^9,10^. Moreover, immune evasion mediated by B2M loss was frequently observed in microsatellite instability (MSI) high tumors, and tumor with B2M loss was more resistant to immune checkpoint blockage (ICB) therapy^11^. The success of anti-tumor immunotherapy has also been proposed to depend on the recognition of the HLA class I complex (heavy chain/B2M/tumor peptide) by CTLs in metastatic melanoma^12^. Recent studies have shown that in addition to the role of B2M in immunity, B2M also widely correlated with proliferation, apoptosis and metastasis of cancer cells^13–16^. Targeting B2M-related signaling pathway shows significant tumor killing activity in a wide range of cancer types, which provides a new strategy for tumor therapy^17–20^.

Based on CRISPR screening, B2M mutation frequently occurred in glioblastoma (GBM)^21^. Specifically, gene disruption of B2M enhanced activity of CAR T cells and resistance to PD-1 inhibition in preclinical model of GBM^22^. However, a comprehensive analysis of B2M in the prognosis and immune microenvironment of gliomas has not yet been reached. In this study, a multidimensional analysis was performed based on public databases to explore the potential relationship between B2M and gliomas, especially LGG. Our findings were investigated in TCGA and verified in CGGA datasets. The upregulation of B2M in gliomas and its relation to an immune suppressive tumor microenvironment indicated that B2M might be a prospective prognostic marker and therapeutic candidate in gliomas.

## Materials and methods

### Data collection and process

We collected glioma samples in The Cancer Genome Atlas (TCGA) containing 672 samples from UCSC Xena (https://xenabrowser.net/) and Chinese Glioma Genome Atlas (CGGA) containing 1013 samples from CGGA website (http://www.cgga.org.cn/). The specific tumor anatomy data of GBM was from Ivy Glioblastoma Atlas Project (http://glioblastoma.alleninstitute.org/). Data about the radiographical regions of normal brain and LGG was from the Gill dataset.

### Bioinformatic analysis

The R project (https://www.r-project.org/) was used for performing all analyses according to gene expression profiles from TCGA and CGGA datasets. In OS, PFI, DSS analysis, the R package Survminer is used to calculate the cut-off point. We downloaded somatic mutations and copy number alternations (CNAs) corresponding with RNA-seq data from TCGA and determined the enrichment of genomic events by GSITIC analysis. We analyzed CNAs related with B2M expression through GISTIC 2.0 analysis (https://gatkforums.broadinstitute.org) with the first 25% and last 25% of samples selected for analysis. We utilized gene sets variation analysis (GSVA) to investigate the immune related biological processes and used ssGSEA algorithm to identify 28 immune cell lineages^23^. MCPcounter was used to identify 10 immune cell lineages^24^. Correlation analysis was conducted according to the expression values of gene ontology (GO) terms and B2M, while selecting the items with p < 0.05 and correlation coefficient > 0.4. Kyoto Encyclopedia of Genes and Genomes (KEGG) database was used for further functional annotation of B2M^25–27^. ESTIMATE (Estimation of Stromal and Immune cells in Malignant Tumor tissues using Expression) algorithm defined immune score, stromal score, and estimate score. B2M related clusters were identified using Partitioning Around Medoid (PAM)^28^. CIBERSORT algorithm was used to quantify 22 immune cell types^29^.

### Statistical analysis

B2M expression differences in tumor characteristics such as WHO grades, MGMT status, 1p19q status, IDH status, and GBM subtypes were analyzed using Wilcoxon rank testing. Spearman correlation analysis was applied to assess the relationship between continual mathematics. Kaplan–Meier survival curves were utilized to depict survival distributions and compared using the log-rank test. Univariate cox regression analyses were utilized to evaluate prognostic value of B2M in pan-cancer samples, which covariates including age and gender were taken into consideration in Cox analysis. Two-way ANOVA analysis followed with Tukey’s analysis was used for comparison of more than two groups. All statistical analyses were performed by R project (version 3.6.1, https://www.r-project.org/), and p-values < 0.05 were considered as significant in all two-sided tests.

## Result

### B2M relates to molecular and clinical features in gliomas

**Figure 1:**
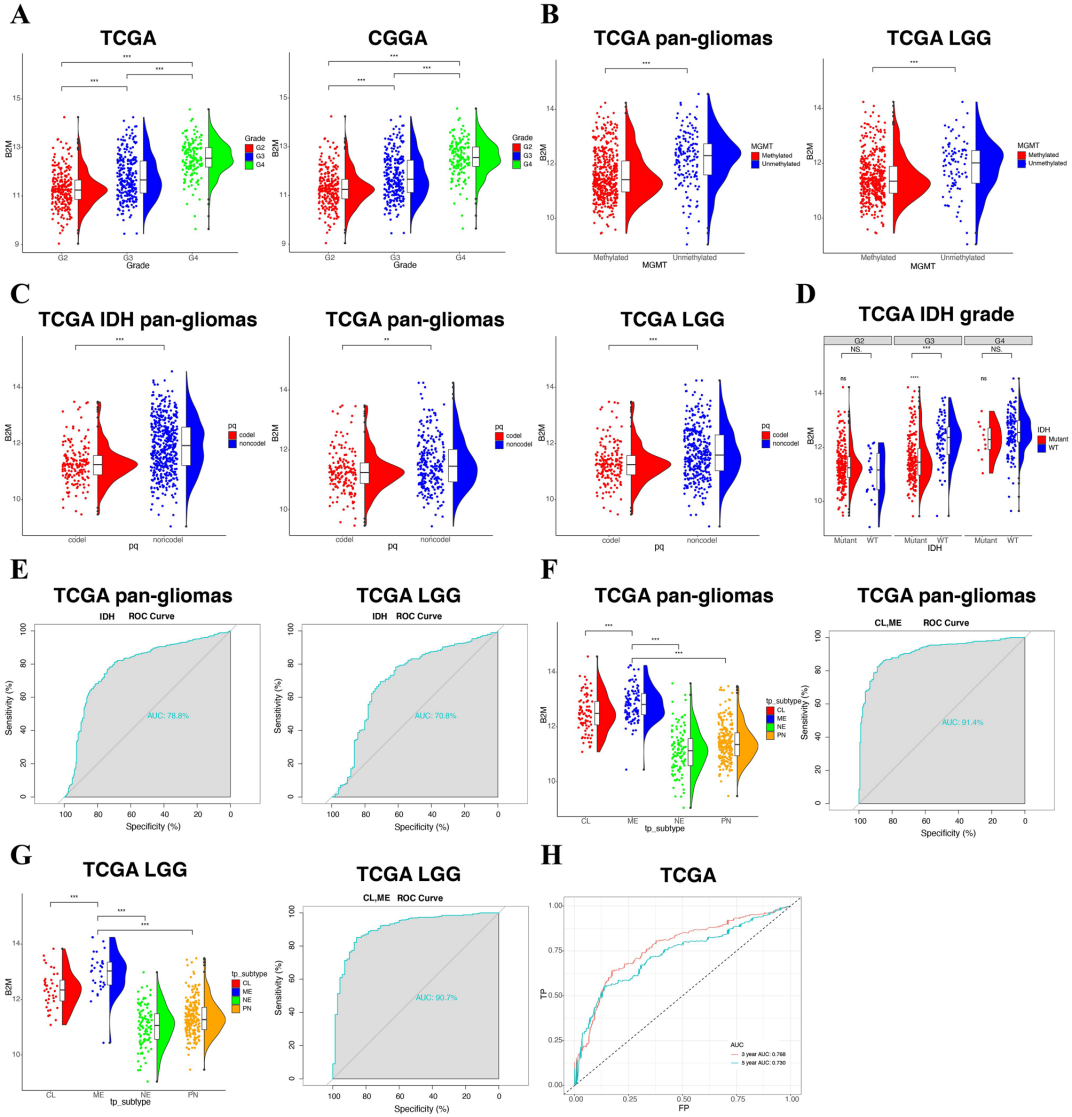
The correlation between B2M expression and diverse characteristics. (**A**) B2M expression in different WHO grades from TCGA and CGGA. (**B**) Association between B2M and MGMT methylation in pan-gliomas and LGG from TCGA. (**C**) Association between B2M and 1p/19q state in pan-gliomas with IDH mutant, pan-gliomas, and LGG samples from TCGA. (**D**) B2M expression in different IDH state from TCGA dataset. (**E**) Receiver operating characteristic (ROC) curve analysis showed that B2M had 78.8% and 70.8% sensitivity and specificity to predict IDH wild-type state gliomas and LGGs, respectively. (**F**) The B2M expression pattern in pan-gliomas from the TCGA molecular subtype. ROC curve analysis showed that B2M had 91.4% sensitivity and specificity to predict CL and ME subtype gliomas. (**G**) The B2M expression pattern in LGGs from the TCGA molecular subtype. ROC curve analysis showed that B2M had 90.7% sensitivity and specificity to predict CL and ME subtype gliomas. (**H**) ROC curve to assess sensitivity and specificity of B2M expression as a diagnostic biomarker in 3 years and 5 years, respectively. *P < 0.05, **P < 0.01, ***P < 0.001. *G2* Grade II, *G3* Grade III, *G4* Grade IV.

Based on the analyses of pan-cancer, B2M expression was highly associated with human primary glioblastoma cell line—U87 cell lineage (Supplementary Fig. S1A) and gliomas samples, including LGG and GBM (Supplementary Fig. S1B). With regard to clinical characteristics, B2M was enriched in recurrent type compared with primary type (Supplementary Fig. S1C). Meanwhile, B2M in progressive disease was significantly higher than in complete remission/response, even though no significance was observed among complete remission/response, partial remission/response, and stable disease (Supplementary Fig. S1D). According to the WHO classification of gliomas, B2M had the highest expression level in GBM samples (Grade IV) in TCGA and CGGA (Fig. 1A). Considered that epigenetic alterations, such as O6-methylguanine DNA methyltransferase (MGMT) methylation, facilitate carcinogenesis^30^, we investigated the correlation between B2M and MGMT methylation and found that B2M was significantly downregulated in methylated samples in pan-gliomas and LGGs (Fig. 1B). B2M was negatively associated with three independent methylation probes with statistical significance (Supplementary Fig. S2A–D). Besides, B2M was significantly enriched in 1p/19q non-codeletion samples (Fig. 1C) and higher histopathologic malignancies (Supplementary Fig. S1E). Isocitrate dehydrogenase (IDH) wild-type gliomas showed upregulated B2M expression with significant difference in LGG samples of grade III (Fig. 1D). In pan-gliomas and LGG samples, the sensitivity and specificity of B2M expression in predicting the IDH wild-type state were high with the area under the curve (AUC) value of 78.8% and 70.8%, respectively (Fig. 1E), which IDH wild-type state has been previously proved with worse outcome and tumor progression in gliomas^31^.

We next analyzed the heterogeneous expression patterns of B2M in gliomas regarding the molecular subtypes, including classical (CL), mesenchymal (ME), pro-neural (PN), and neural (NE), with CL and ME being the more aggressive subtypes^32,33^. As shown in Fig. 1F,G, B2M was more upregulated in CL and ME subtypes in pan-gliomas and LGGs compared to PN and NE subtypes. Surprisingly, the AUC value of B2M expression in predicting CL and ME subtypes were 91.4% and 90.7% in pan-gliomas and LGGs, respectively, indicating that B2M effectively predicted the more aggressive subtypes. According to the transcriptomic data from Ivy Glioblastoma Atlas Project, B2M was mainly enriched in hyperplastic blood vessels and microvascular proliferation (Supplementary Fig. S1F).

Moreover, B2M expression sensitively and specifically predicted 3 years and 5 years survival with the AUC value of 76.8% and 73.0%, respectively (Fig. 1H). The above findings indicated that B2M predicted a more aggressive glioma subtypes and served as a crucial part in the tumorigenic process of gliomas.

### B2M predicts worse clinical outcomes in gliomas

**Figure 2:**
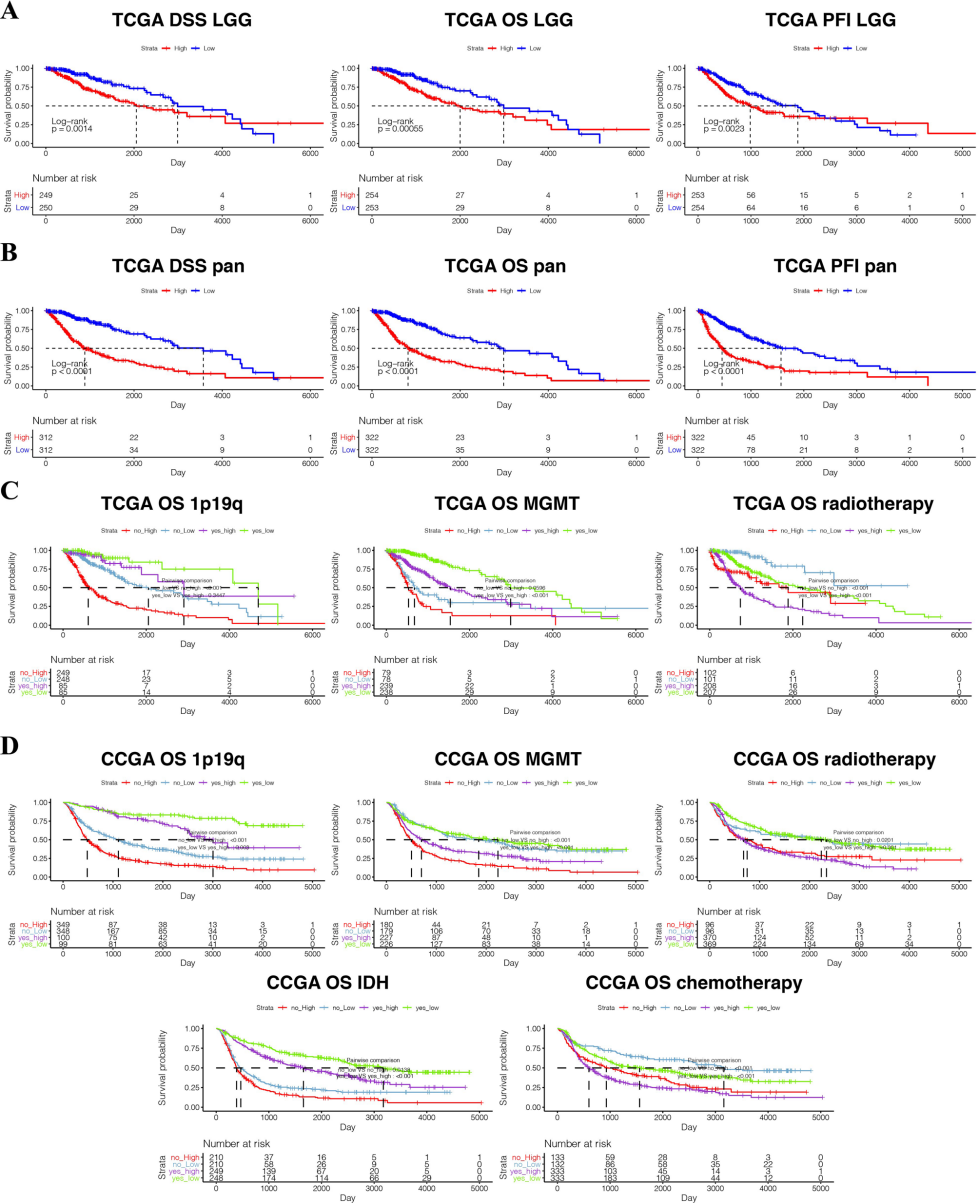
B2M expression is associated with poor survival in glioma patients. Kaplan–Meier analysis of overall survival (OS), disease specific survival (DSS), and progression-free interval (PFI) based on B2M expression were performed in LGGs (**A**) and pan-gliomas (**B**) from TCGA. Kaplan–Meier analysis of OS based on B2M expression in different prognostic factors including 1p19q, MGMT, radiotherapy, IDH, chemotherapy was performed in TCGA (**C**) and CGGA (**D**).

**Figure 3:**
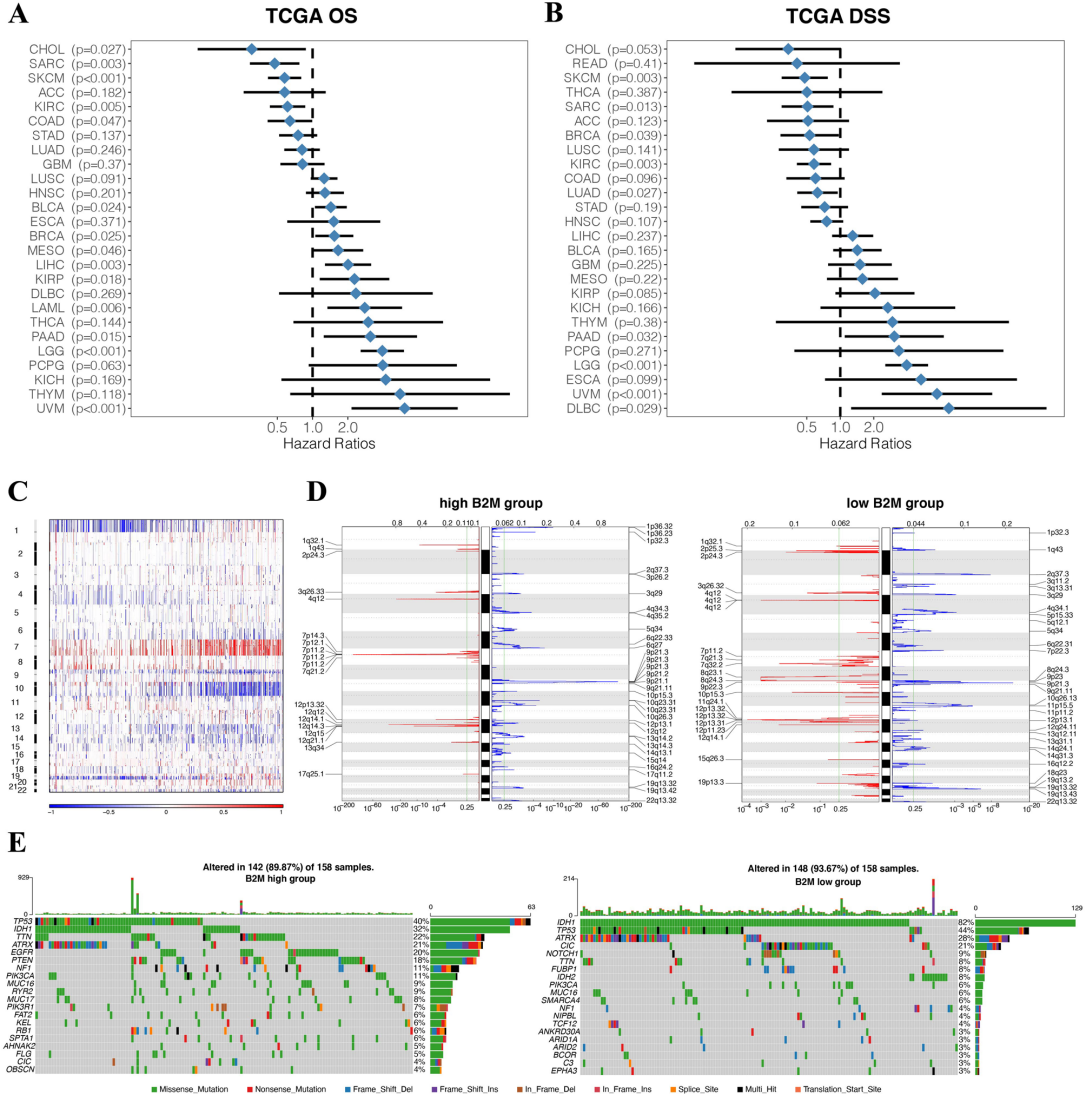
Distinct genomic profiles correlated with B2M expression. Univariate cox regression analyses estimating prognostic value of B2M in OS (**A**) and DSS (**B**) in different cancer types from TCGA. The length of horizontal line represents the 95% confidence interval for each group. The vertical dotted line represents the HR of all patients. HR < 1.0 indicates that high TME score is a favorable prognostic biomarker. Number of patients is indicated. *TGCT* testicular germ cell tumors, *THYM* thymoma, *UVM* uveal melanoma, *READ* rectum adenocarcinoma, *LGG* brain lower grade glioma, *PAAD* pancreatic adenocarcinoma, *KICH* kidney chromophobe, *LUSC* lung squamous cell carcinoma, *ESCA* esophageal carcinoma, *STAD* stomach adenocarcinoma, *LUAD* lung adenocarcinoma, *GBM* glioblastoma multiforme, *HNSC* head and neck squamous cell carcinoma, *OV* ovarian serous cystadenocarcinoma, *BRCA* breast invasive carcinoma, *COAD* colon adenocarcinoma, *LIHC* liver hepatocellular carcinoma, *BLCA* bladder urothelial carcinoma, *UCEC* uterine corpus endometrial carcinoma, *KIRC* kidney renal clear cell carcinoma, *LAML* acute myeloid leukemia, *KIRP* kidney renal papillary cell carcinoma, *SARC* sarcoma, *CESC* cervical squamous cell carcinoma and endocervical adenocarcinoma, *MESO* mesothelioma, *SKCM* skin cutaneous melanoma, *UCS* uterine carcinosarcoma, *CHOL* cholangiocarcinoma, *PRAD* prostate adenocarcinoma, *DLBC* lymphoid neoplasm diffuse large B-cell lymphoma, *THCA* thyroid carcinoma, *PCPG* pheochromocytoma and paraganglioma. P-values were obtained from the log-rank test. (**C**) The overall CNAs profile arranged by high and low B2M expression. Blue (deletion); red (amplification). (**D**) Frequency of amplifications and deletions generated by GISTIC2.0 analysis and stratified by B2M expression in gliomas. Deletion is blue and amplification is red. (**E**) Distinct somatic mutations in gliomas.

Subsequently, we investigated the prognostic value of B2M in human gliomas using Kaplan–Meier analysis. In pan-gliomas and LGGs from TCGA dataset, disease-specific survival (DSS), overall survival (OS), and progression-free interval (PFI) of patients with high B2M were significantly shorter than those with low B2M (Fig. 2A,B). Survival curves were also depicted based on 2016 WHO molecular classification (Supplementary Fig. S3). Glioma patients with high B2M expression had worse prognosis in several molecular subgroups. We also evaluated the prognostic value of B2M in combination with five prognostic factors including 1p19q, MGMT, IDH, chemotherapy, and radiotherapy (Fig. 2C,D). B2M showed worse prognosis when glioma patients were stratified by the five clinical prognostic factors (Fig. 2C,D). In pan-cancer samples, B2M predicted worse OS (Supplementary Fig. S4) and DSS (Supplementary Fig. S5) in Bladder Urothelial Carcinoma (BLCA, n = 405), Kidney renal clear cell carcinoma (KIRC, n = 528), Liver hepatocellular carcinoma (LIHC, n = 363), Lung squamous cell carcinoma (LUSC, n = 491), Thyroid carcinoma (THCA, n = 503), Thymoma (THYM, n = 118), Uveal Melanoma (UVM, n = 79), Pancreatic adenocarcinoma (PAAD, n = 177), Sarcoma (SARC, n = 258), Skin Cutaneous Melanoma (SKCM, n = 101), and Testicular Germ Cell Tumors (TGCT, n = 132). Moreover, in terms of OS, B2M was a hazardous factor in 9 independent cancer types and a favorable factor in 5 independent cancer types with statistical significance of p < 0.05 (Fig. 3A). Likewise, B2M was a hazardous factor in 4 independent cancer types and a favorable factor in 5 independent cancer types in terms of DSS with statistical significance of p < 0.05 (Fig. 3B). In sum, B2M can predict the poor prognosis of gliomas.

### B2M correlates with diverse genomic alteration patterns

To explore the relationship between B2M and genomic profiles in gliomas, somatic mutation analysis was carried out. We observed a global CNV profile based on contrast of the clusters with high expression level of B2M expression (n = 158) and low expression level of B2M (n = 158) (Fig. 3C,D). The cluster with high B2M expression exhibited chr7 amplification and chr10 deletion, two vital genomic features in GBM^34^ (Fig. 3C). Deletion of 1p and 19q were more frequent in cluster with low B2M expression (Fig. 3C). Cluster with high B2M expression showed more amplified peaks in PDGFRA (4q12), EGFR (7p11.2) and CDK4 (12q14.1), all of which are oncogenic driver genes (Fig. 3D). Cluster with high B2M expression also showed deleted peaks in CDKN2A/CDKN2B (9p21.3) and PTEN (10q23.3) (Fig. 3D). More frequent mutations in IDH1 (32%), TTN (22%), ATRX (21%), EGFR (20%), and PTEN (18%) were observed in the cluster with high B2M expression, while more frequent mutations in IDH1 (82%), ATRX (28%), CIC (21%) occurred in cluster with B2M low expression (Fig. 3E). Moreover, gliomas with CN loss showed low expression of B2M (Supplementary Fig. S6). Thus, these results showed that whole chromosomal variations might have potential influence on B2M expression.

### B2M correlates with immune and stromal signatures

Infiltrating stromal cells and immune cells mediate tumor signaling^35,36^. Therefore, we explored the relationship between B2M and the ESTIMATE score. In pan-glioma (Supplementary Fig. S7A) and LGG (Supplementary Fig. S7B) samples, B2M positively correlated with immune, stromal and ESTIMATE scores, and the correlation was significant. 28 kinds of immune cells and stromal cells were identified for further establishing the relationship between B2M and tumor microenvironment. B2M positively correlated with multiple infiltrating immune cell types, such as NK cells, CD4+ T effector memory cells (TEM), and CD8+ TEM, which play a critical role in anti-tumor response. B2M also positively correlated with immunosuppressive cells including MDSCs, regulatory T cells (Treg), macrophages, mast cells, and monocytes (Supplementary Fig. S7C).

Moreover, 10 immune cell types were identified, and B2M highly correlated with stromal cells, such as epithelial cells and fibroblasts^37^ (Supplementary Fig. S8A–C). Taken together, B2M could potentially mediate immune and stromal cell infiltration in gliomas microenvironment. CIBERSORT algorithm was further used to quantify 22 immune cell types. The expression differences of B2M in the 22 immune cells types were explored in TCGA (Supplementary Fig. S9A) and CGGA (Supplementary Fig. S9B). Generally, high B2M expression was observed in a majority of immune cells.

### B2M is involved in immune related processes

**Figure 4:**
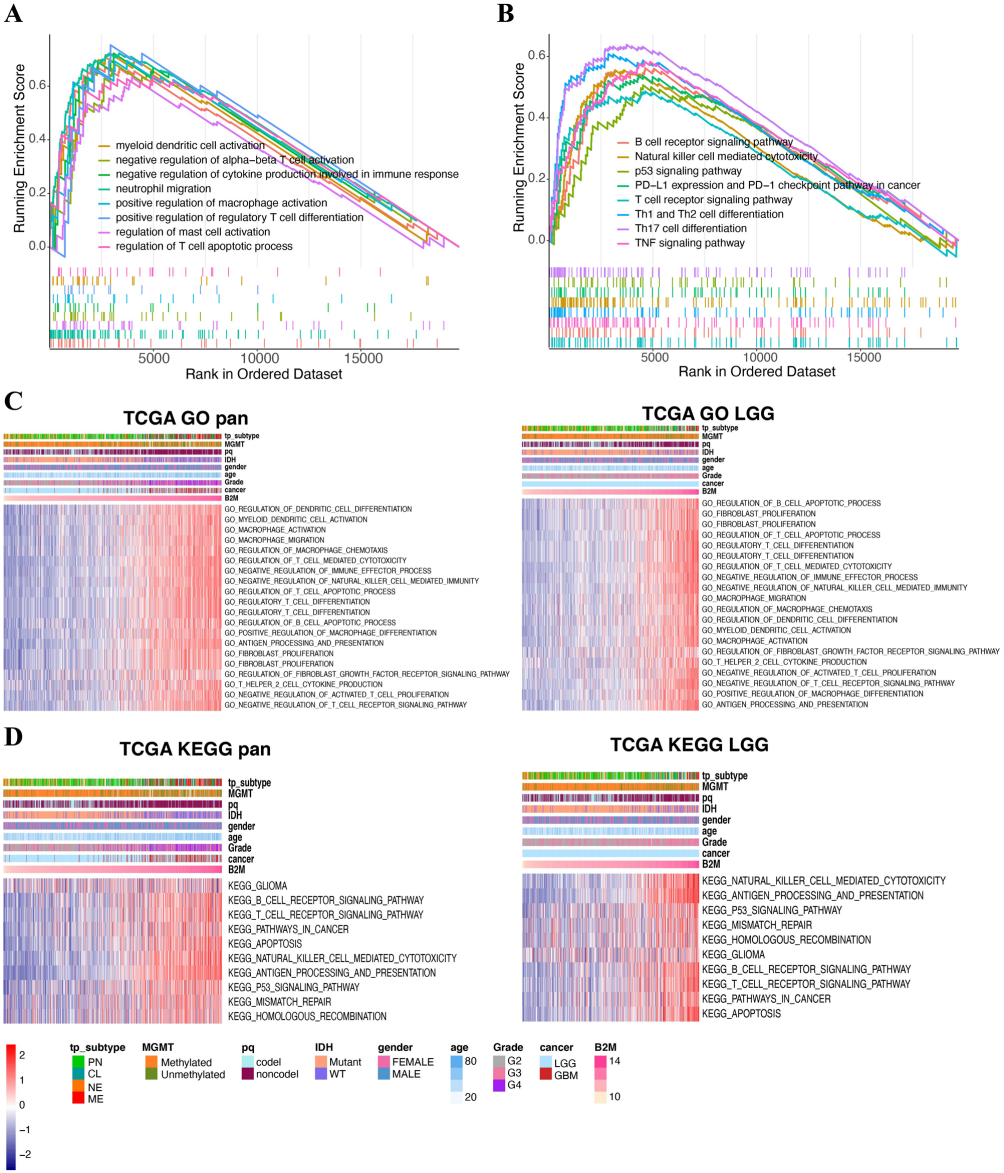
B2M-related immune functions in gliomas. (**A**,**B**) GSEA plots for enrichment of immunogenic and oncogenic signaling pathways from the GO (**A**) and KEGG (**B**) database^25–27^, respectively. (**C**,**D**) The relationship between B2M and biological processes in pan-gliomas and LGGs from TCGA dataset. Results are based on the GO (**C**) and KEGG (**D**) databases^25–27^, respectively. Expression values of B2M are z-transformed and are colored red for high expression and blue for low expression, as indicated in the scale bar.

GO and KEGG analysis were performed to explore biological roles of B2M in the occurrence and development of human gliomas. GSEA with all transcripts showed that B2M was associated with diverse immune-related pathways in GO (Fig. 4A) and KEGG (Fig. 4B) analysis, respectively. In addition, GO enrichment results (Supplementary Fig. S10A) also indicated that B2M had diverse influences on immune related biological processes, including neutrophil activation, T cell mediated cytotoxicity, regulation of lymphocyte activation, regulation of T cell activation, and macrophage activation. These results were verified in TCGA and CGGA datasets (Fig. 4C and Supplementary Fig. S10C). KEGG analysis also revealed the robust relationship between B2M and immune-related pathways, including TNF signaling, Th1 and Th2 cell differentiation (Supplementary Fig. S10B). In both CGGA and TCGA cohorts, B2M was associated with natural killer cell mediated cytotoxicity, apoptosis, T cell and B cell receptor signaling pathway (Fig. 4D and Supplementary Fig. S10D). These results proved that B2M positively participated in diverse immune related pathways in gliomas.

### B2M shows a robust relationship with immune checkpoint molecules

**Figure 5:**
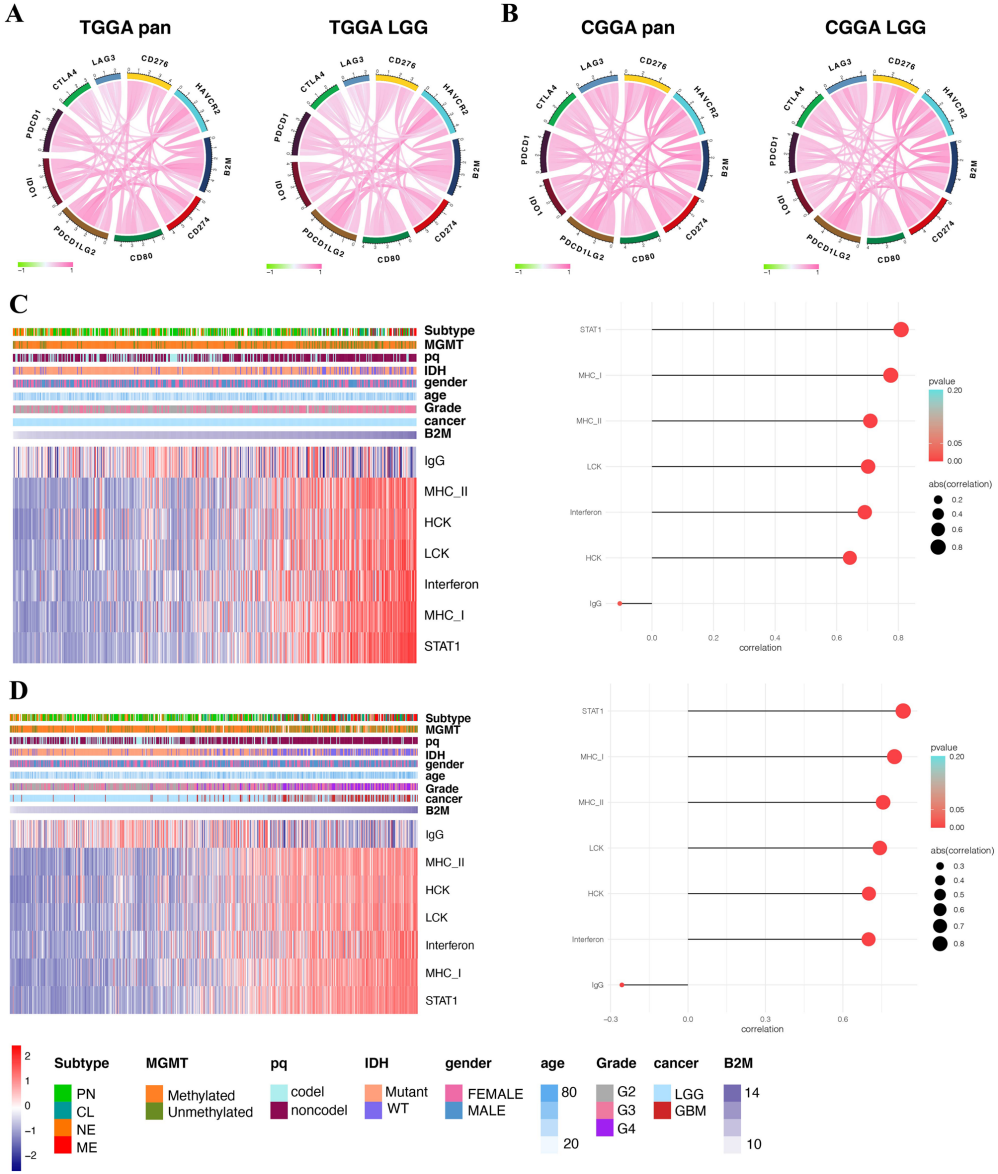
B2M correlates with immune checkpoint members and inflammatory activities in gliomas. B2M is relevant to other immune checkpoint molecules in pan-gliomas and LGGs from TCGA (**A**) and CGGA (**B**). Heatmaps illustrate the relationship between B2M and inflammatory activities in LGGs (**C**) and pan-gliomas (**D**) from TCGA. Expression values of B2M are z-transformed and are colored red for high expression and blue for low expression, as indicated in the scale bar.

Considering the increasing clinical benefits of immune checkpoint blockage^38,39^, we evaluated the relationship between B2M and classical immune checkpoint molecules. B2M exhibited a highly positive correlation with immune checkpoint molecules, such as PDCD1LG2, HAVCR2, CD274 in pan-gliomas and LGG samples, respectively (Fig. 5A,B). As mentioned above, tumor with high B2M expression had increased MHC-I-based antigen presentation and associated CD8 cytolytic responses, which gliomas were likely to adopt immune evasive mechanisms through expression of immune checkpoint molecules. We speculated that B2M might potentially regulate immunosuppression via the in-depth interplay with immune checkpoint molecules in tumor microenvironment in gliomas.

### B2M participates in inflammatory activities

Previous research has proved that B2M was associated with inflammation in breast cancer^40^. The relationship between B2M and seven inflammatory activity related signatures was then explored in pan-gliomas (Fig. 5C) and LGG (Fig. 5D), B2M expression had positive correlation with HCK, LCK, MHC-I, MHC-II, STAT1 and interferon metagenes, and negative correlation with IgG metagene expression. Thus, B2M could probably be enriched in macrophage activation, T cell activation, and antigen presentation.

### Construction of B2M related clusters of glioma patients

**Figure 6:**
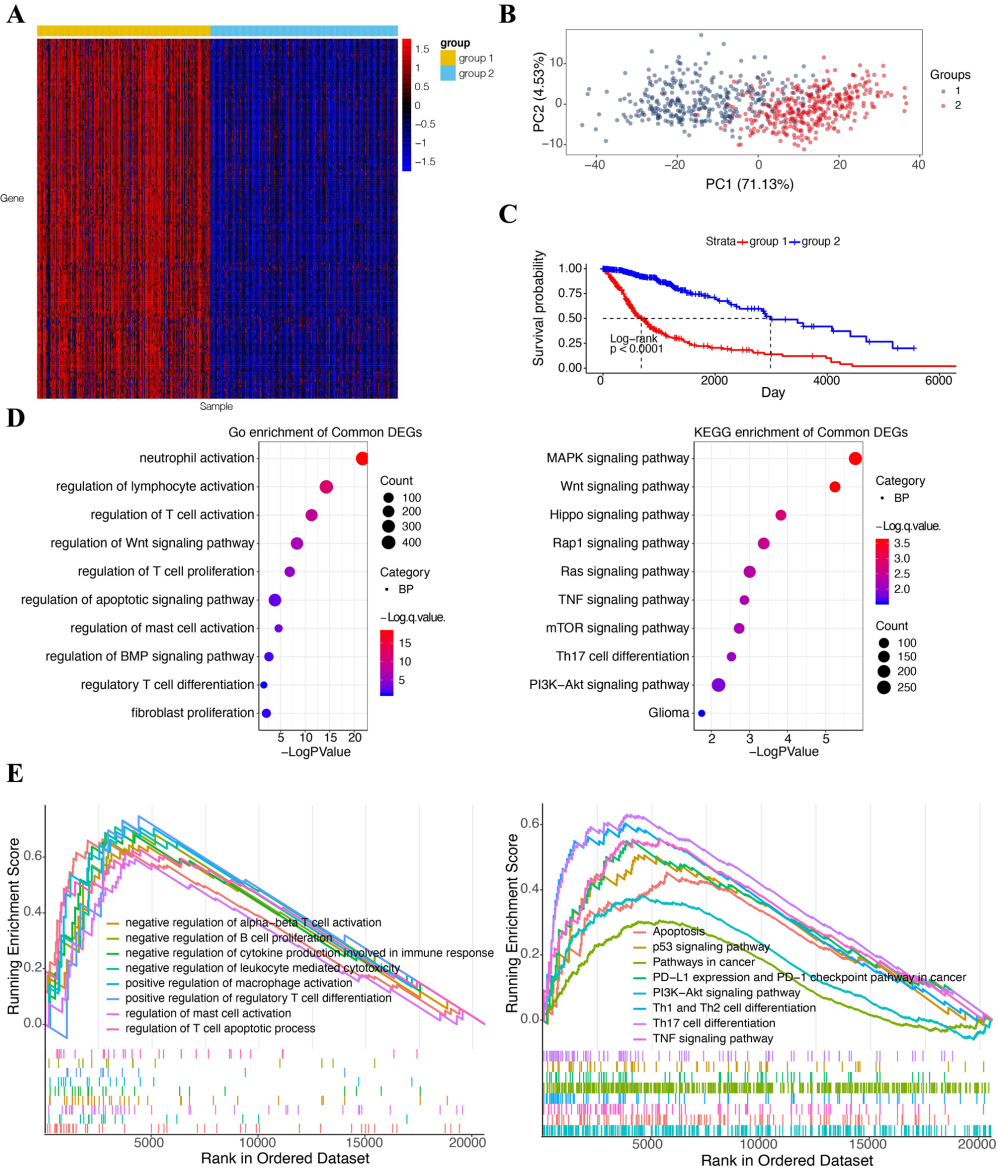
Classification of glioma patients and characteristics of each subtypes in TCGA cohort. (**A**) Heatmaps illustrate the condition of classification. Each column represents the glioma sample and each row represents the B2M-related gene. Expression values of B2M-related genes are z-transformed and are colored red for high expression and blue for low expression, as indicated in the scale bar. (**B**) Principal component analysis (PCA) shows the consequence of classification. (**C**) Kaplan–Meier analysis of OS was performed in two clusters. (**D**) GO and KEGG enrichment analysis of two clusters relevant signature genes^25–27^. (**E**) GSEA of two clusters relevant signature genes sets downloaded from the GO and KEGG database, respectively^25–27^.

Further, we explored the prognostic value of B2M by assessing its ability in distinguishing glioma patients. To choose the best cluster number, we utilized the ConsensusClusterPlus software to evaluate the clustering stability, supporting the presence of two glioma subtypes (Supplementary Fig. S11A–D). We selected 189 B2M-related genes in TCGA, of which correlation efficient > 0.7 or <  − 0.7. Cluster analysis based on the 189 identified genes showed two different patterns of B2M-related genes in glioma patients from TCGA (Fig. 6A). PCA analysis based on B2M-related genes showed two clearly separated glioma patient groups from TCGA (Fig. 6B). Group 1 showed worse prognosis compared to group 2 (Fig. 6C). GO and KEGG enrichment analysis (Fig. 6D) of these differentially expressed genes (DEGs) was conducted using the R package clusterProfiler, indicating the association between DEGs and immune activations. Using GSEA in GO and KEGG, we found DEGs were involved in immunosuppressive processes and tumor related pathway (Fig. 6E). These results indicated that B2M was a potential therapeutic candidate in gliomas and confirmed that B2M correlated with immune related processes and tumor development.

## Discussion

B2M, comprising the light chain of MHC-I molecules, regulates cancer immune escape and metastatic progression^12^. With more and more attention being paid to the regulatory role of B2M in various cancer types, B2M is considered as a potential mediator for improving the treatment strategy. Since the expression pattern of B2M in glioma specimens is not clear, we used large-scale bioinformatics analysis to study the distribution of B2M in gliomas. Here, our data showed that the up-regulated B2M expression was observed in LGG samples, especially in samples with IDH wild-type based on 2016 WHO classification. B2M was significantly downregulated in methylated samples in pan-gliomas and LGGs. B2M expression was also mainly observed in hyperplastic blood vessels and microvascular proliferation. In addition, B2M was highly enriched in CL and ME subtypes of gliomas and could be used as a predictor with high sensitivity for gliomas. Notably, ME subtypes are rich in immunosuppressive cytokines and immunosuppressive checkpoint inhibitors, which are characterized by immunosuppression and aggression^32,33,41^. Taken together, B2M was associated with the malignancy of gliomas.

B2M is abnormally expressed in many cancer types. Plenty of researches have shown that the concentration of B2M in serum or urine raised in diverse diseases, such as breast cancer, prostate cancer, lung cancer, renal cancer, multiple myeloma, and especially non-Hodgkin's lymphoma^42–47^. Importantly, our results also showed the negative correlation between B2M expression and patient survival in TCGA and CGGA databases. Further, B2M was significantly associated with five prognosis factors including IDH, 1p19q, MGMT, chemotherapy, and radiotherapy in multivariable survival analysis. In patients stratified by the five prognosis factors, B2M predicted worse survival. These results suggest that B2M was widely involved in the carcinogenic process of gliomas and specifically predicted the poor prognosis of glioma patients.

When analyzing the unique genomic alternation, B2M expression was observed with a positive correlation with somatic mutations and CNA. In samples with high B2M expression, oncogenic drivers, including PDGFRA, EGFR and CDK4 were frequently amplified genomic peaks. Besides, we observed the deletion peaks of tumor suppressive genes such as CDKN2A/CDKN2B and PTEN. Importantly, the alternation and heterogeneity of the genome has a positive impact on the transformation of the tumor proliferation, tumor progression, tumor microenvironment, and treatment resistance^48^. These findings revealed that B2M was probably related to malignant biological processes.

We are the first to explore the relationship between B2M and ESTIMATE scores. B2M had obviously positive correlation with ESTIMATE scores. The positive correlation between B2M and infiltrating immune cells, stromal cells (including DC, MDSC, TEM, Treg, macrophages, mast cells, neutrophils, NK cells, monocytes) were also observed. In addition, GSVA analysis suggested that B2M suppressed the anti-tumor immune response associated with T cells. These results suggested that B2M had an effect on the establishment of an immunosuppressive microenvironment in gliomas. In B2M related biological processes, GSEA and GSVA showed that B2M was involved in immune-related pathways, such as myeloid dendritic cell activation, T cell mediated cytotoxicity, and macrophage activation in GO analysis. While in KEGG analysis, B2M was associated with natural killer cell mediated cytotoxicity, and apoptosis. These results showed that B2M may be essential to immune processes of gliomas.

Because immune checkpoint blockage has demonstrated remarkable results in cancer treatment, combinations of immune checkpoint inhibitors play an important role for melanoma and brain metastases patients by enhancing their response rates and longer survival^49–52^. In colorectal cancer (CRC), B2M mutation was proposed to reshape the microsatellite-unstable (MSU) CRC for resistance of ICB treatment^53^. In melanoma, B2M mutation was also associated with resistance of T cell and PD-1 blockage^54,55^. Our results showed that B2M had a high correlation with PDCD1, CD274, PDCD1LG2, CD276, CTLA-4, IDO1, HAVCR2, and CD80 in pan-glioma and LGG. Notably, B2M potentially participated in inflammatory activities as B2M positively correlated with MHC-I, MHC-II, STAT1 and interferon. The high correlation between MHC-I and B2M expression further confirmed the B2M-induced MHC-I-based antigen presentation forced gliomas to express more immune checkpoint molecules to escape immune surveillance. Therefore, the high expression of immune checkpoint molecules might represent an immune suppressive microenvironment that was more likely to exist in gliomas with high B2M expression. It should be noted that MHC-I formed by B2M plays a central role in the immuno-evasion mechanism of immune checkpoint blockade, which MHC-I expression has been proved to predict checkpoint blockade response^56,57^. Thus, inhibitors targeting the B2M in combination with other immune checkpoint molecules may revolutionize the treatment of gliomas. However, experimental studies were essentially needed for further validation of its therapeutic value and regulatory role in immune related responses in gliomas before any attempt to target B2M in a clinical setting.

To sum up, based on our bioinformatics analysis, we confirmed that B2M, as a molecular target, played a potential role in the anti-cancer treatment of glioma. B2M was positively related with the high malignant degree of glioma and the poor survival of patients. Additionally, B2M potentially participated in the inflammatory response in the glioma microenvironment, interacted with other immune checkpoint molecules, and suppressed the anti-tumor immunity. The role of B2M in tumor microenvironment and immune regulation need to be further elucidated by experiments.

## Supplementary Information

Supplementary Information.

## Data Availability

The datasets generated and analyzed during the current study are available in the TCGA data source (https://xena.ucsc.edu) and CGGA data portal (http://www.cgga.org.cn).
