# Supplementary material for: B2M overexpression correlates with malignancy and immune signatures in human gliomas
Source: Sci Rep. 2021 Mar 3;11:5045. doi: 10.1038/s41598-021-84465-6 (PMC7930032; doi:10.1038/s41598-021-84465-6)
Supplement: Supplementary file 1 — Supplementary Information. [file 41598_2021_84465_MOESM1_ESM.docx]

**B2M overexpression correlates with** **malignancy and immune signatures in human gliomas**

Hao Zhang^1#^, Biqi Cui^6#^, Yulai Zhou^4,5^, Xinxing Wang^1^, Wantao Wu^4,5^, Zeyu Wang^1^, Ziyu Dai^1^, Kui Yang^1*^, Cheng quan^1,2,3*^

^1^ Department of Neurosurgery, Xiangya Hospital, Central South University, Changsha, China

^2^ Department of Clinical Pharmacology, Xiangya Hospital, Central South University, Hunan, China

^3^ Center for Medical Genetics & Hunan Provincial Key Laboratory of Medical Genetics, School of Life Sciences, Central South University, Changsha, China

^4^ Department of Oncology, Xiangya Hospital, Central South University, Changsha, Hunan 410008, China

^5^ National Clinical Research Center for Geriatric Disorders, Xiangya Hospital, Central South University, Changsha, Hunan 410008, China

^6^ Department of Neurology, Xiangya Hospital, Central South University

^#^Equal contribution

*Corresponding authors:

Quan Cheng, Department of Clinical Pharmacology, Xiangya Hospital, Central South University, Address: Changsha 410008, Hunan, P. R. China. E-mail: [chengquan@csu.edu.cn](mailto:chengquan@csu.edu.cn). Telephone: +86-731-89753037; Fax: +86-731-84327401

Kui Yang, Department of Neurosurgery, Xiangya Hospital, Center South University, Address: Changsha 410008, Hunan, P. R. China. E-mail: [yangkui@csu.edu.cn](mailto:yangkui@csu.edu.cn). Telephone: +86-731-89753037; Fax: +86-731-84327401

**Running title:** B2M correlates with malignancy in glioma


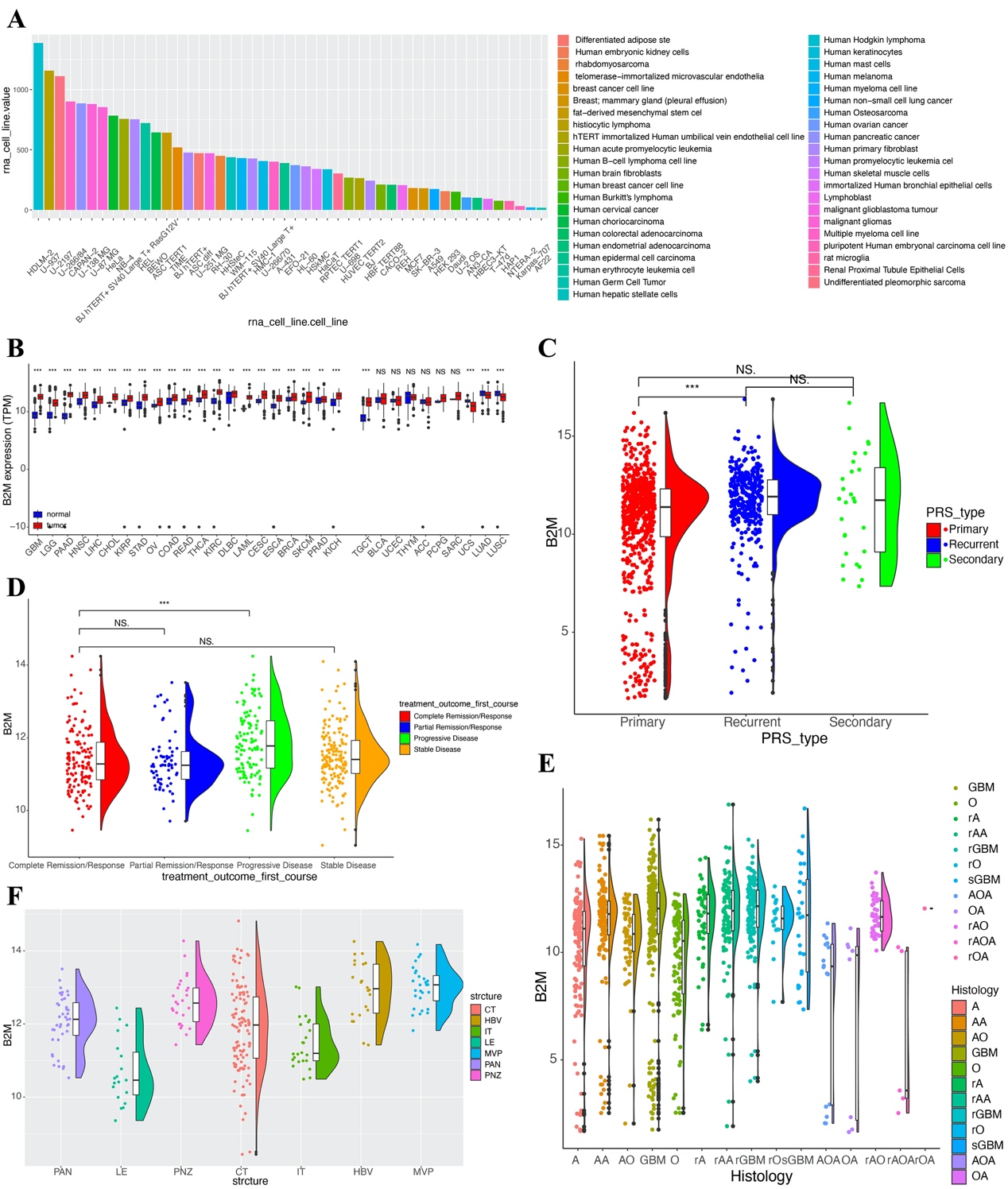
**Figure S1.** Clinical and molecular features in associations with B2M expression. **A.** B2M expression in different cell lines. **B.** B2M expression in different tumors. GBM, Glioblastoma multiforme; LGG, Brain Lower Grade Glioma; PAAD, Pancreatic adenocarcinoma; HNSC, Head and Neck squamous cell carcinoma; LIHC, Liver hepatocellular carcinoma; CHOL, Cholangiocarcinoma; KIRP, Kidney renal papillary cell carcinoma; STAD, Stomach adenocarcinoma; OV, Ovarian serous cystadenocarcinoma; COAD, Colon adenocarcinoma; READ, Rectum adenocarcinoma; THCA, Thyroid carcinoma; KIRC, Kidney renal clear cell carcinoma; DLBC, Lymphoid Neoplasm Diffuse Large B-cell Lymphoma; LAML, Acute Myeloid Leukemia; CESC, Cervical squamous cell carcinoma and endocervical adenocarcinoma; ESCA, Esophageal carcinoma; BRCA, Breast invasive carcinoma; SKCM, Skin Cutaneous Melanoma; PRAD, Prostate adenocarcinoma; KICH, Kidney Chromophobe; TGCT, Testicular Germ Cell Tumors; BLCA, Bladder Urothelial Carcinoma; UCEC, Uterine Corpus Endometrial Carcinoma; THYM, Thymoma; ACC, Adrenocortical carcinoma; PCPG, Pheochromocytoma and Paraganglioma; SARC, Sarcoma; UCS, Uterine Carcinosarcoma; LUAD, Lung adenocarcinoma; LUSC, Lung squamous cell carcinoma. **C.** B2M expression in primary, recurrent, and secondary types. **D.** B2M expression in different treatment outcomes. **E.** B2M expression in different histopathologic classification from CGGA dataset. A, low-grade astrocytoma; AA, anaplastic astrocytoma; AO, anaplastic oligodendroglioma; GBM, glioblastoma; O, oligodendroglioma; rA, recurrent low-grade astrocytoma; rAA, recurrent anaplastic astrocytoma; rGBM, recurrent glioblastoma; rO, recurrent oligodendroglioma; sGBM, secondary glioblastoma; AOA, anaplastic oligoastrocytoma; OA, oligoastrocytoma. **F.** Intra-tumor analysis of PDIA3 expression. LE (Leading Edge), IT (Infiltrating Tumour), CT (Cellular Tumour), PAN (Pseudopalisading Cells Around Necrosis), PNZ (Perinecrotic Zone), MVP (Microvascular Proliferation), and HBV (Hyperplastic Blood Vessels). NS, *, **, and *** indicate p < .05, P < .01, p < .001, and no significant difference, respectively.


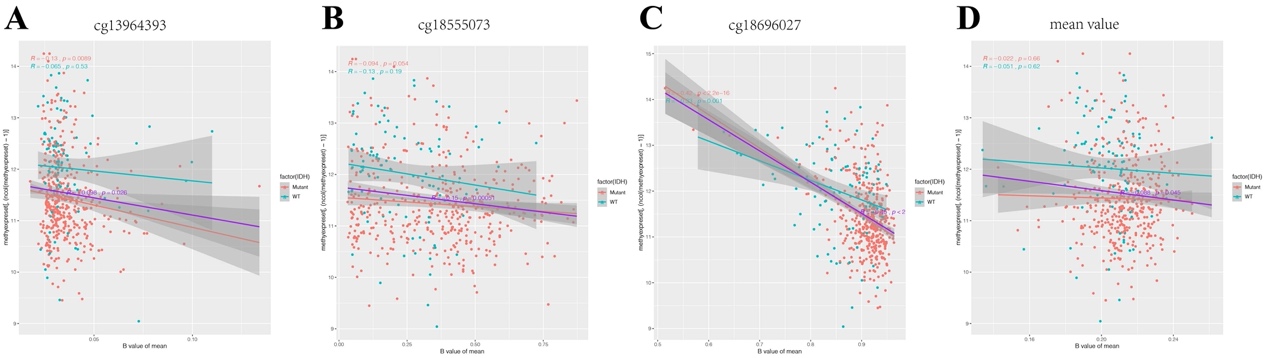


**Figure S2.** Relationship between B2M and methylation status at promoter region in LGG samples from TCGA: (A) cg13964393 loci; (B) cg18555073 loci; (C) cg18696027 loci; (D) mean value of methylation status. The orange dots indicate IDH‐mutant samples, and cyan dots indicate IDH wild‐type samples, respectively. The orange line and cyan line indicate linear regression between B2M expression and promoter region methylation in IDH‐mutant samples and IDH wild‐type samples, respectively. The purple line represents the linear regression between B2M expression and promoter region methylation regardless of IDH mutation status.


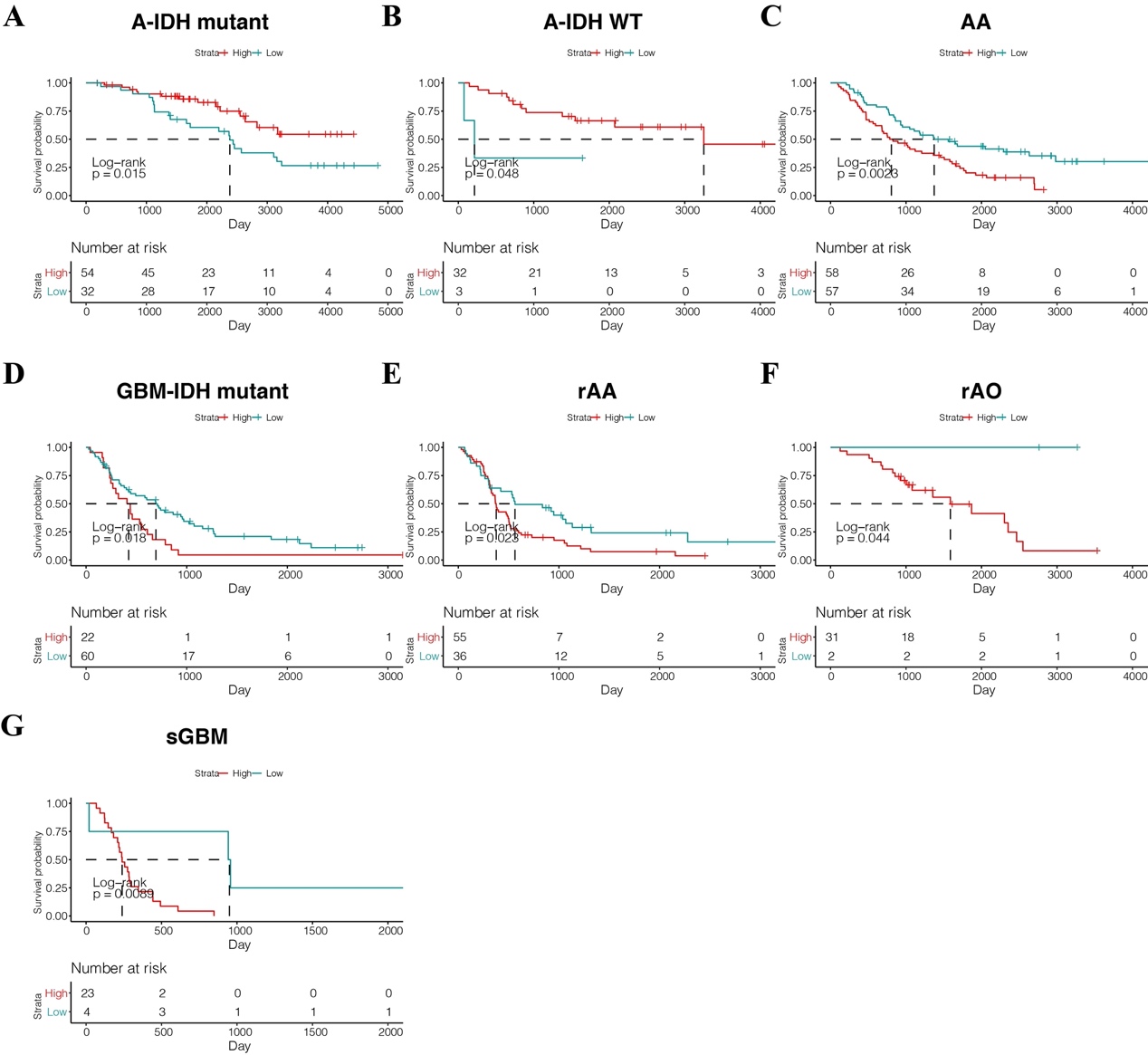


**Figure S3.** Prognostic value of the risk signature in patients stratified by the integrated analysis of WHO 2016. (**A-G)** Kaplan-Meier overall survival (OS) curves for patients with low-grade astrocytoma with IDH-mutant (**A**), low-grade astrocytoma with IDH wide type (**B**), anaplastic astrocytoma (**C**), glioblastoma with IDH mutant (**D**), recurrent anaplastic astrocytoma (**E**), recurrent anaplastic oligodendroglioma (**F**), secondary glioblastoma (**G**).


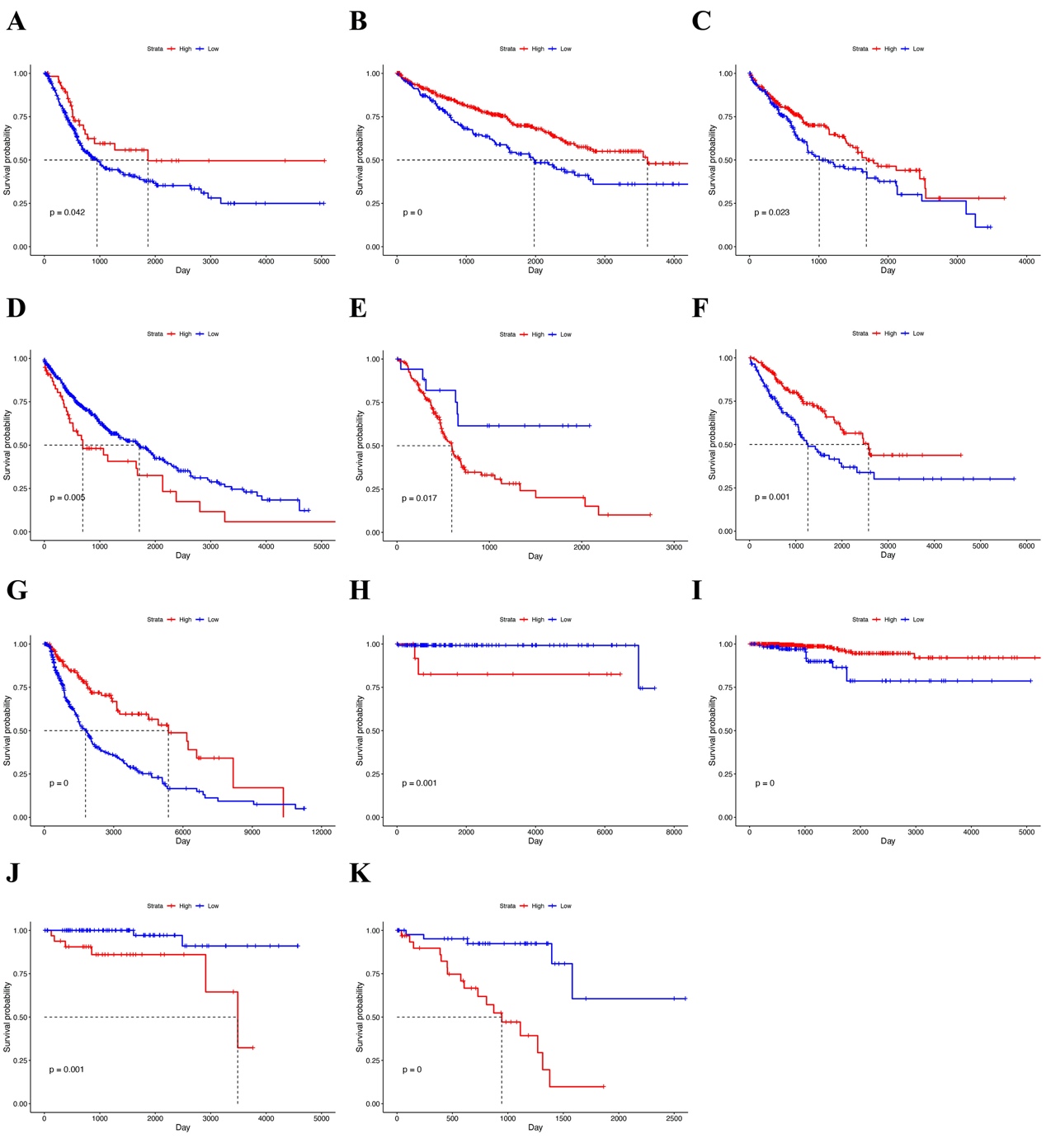


**Figure S4.** Kaplan-Meier analysis using high vs low B2M expression for OS in different cancer. **A.** BLCA, Bladder Urothelial Carcinoma; **B.** KIRC, Kidney renal clear cell carcinoma; **C.** LIHC, Liver hepatocellular carcinoma; **D.** LUSC, Lung squamous cell carcinoma; **E.** PAAD, Pancreatic adenocarcinoma; **F.** SARC, Sarcoma; **G.** SKCM, Skin Cutaneous Melanoma; **H.** TGCT, Testicular Germ Cell Tumors; **I.** THCA, Thyroid carcinoma; **J.** THYM, Thymoma; **K.** UVM, Uveal Melanoma.


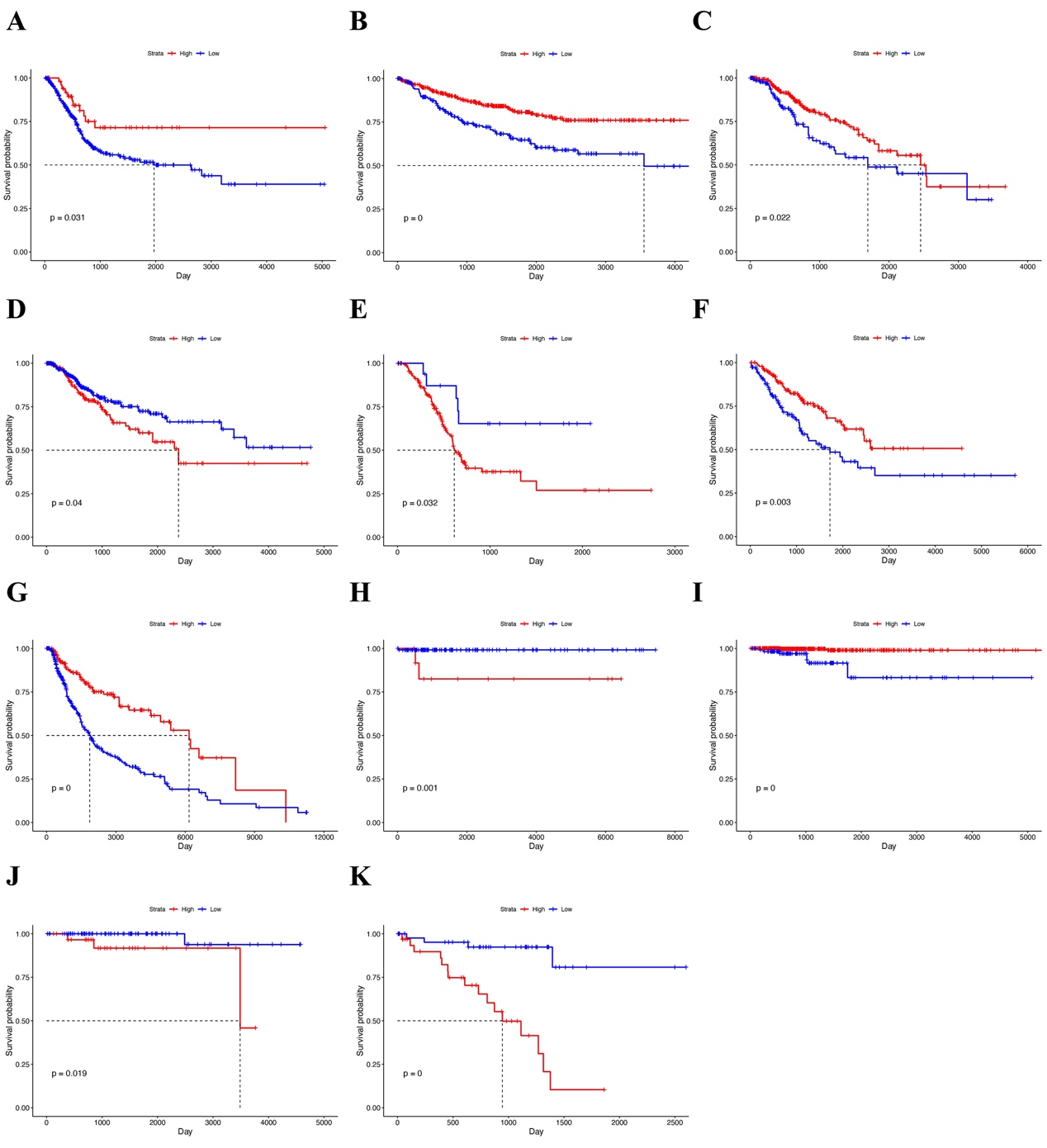


**Figure S5.** Kaplan-Meier analysis using high vs low B2M expression for disease specific survival (DSS) in different cancer. **A.** BLCA, Bladder Urothelial Carcinoma; **B.** KIRC, Kidney renal clear cell carcinoma; **C.** LIHC, Liver hepatocellular carcinoma; **D.** LUSC, Lung squamous cell carcinoma; **E.** PAAD, Pancreatic adenocarcinoma; **F.** SARC, Sarcoma; **G.** SKCM, Skin Cutaneous Melanoma; **H.** TGCT, Testicular Germ Cell Tumors; **I.** THCA, Thyroid carcinoma; **J.** THYM, Thymoma; **K.** UVM, Uveal Melanoma.


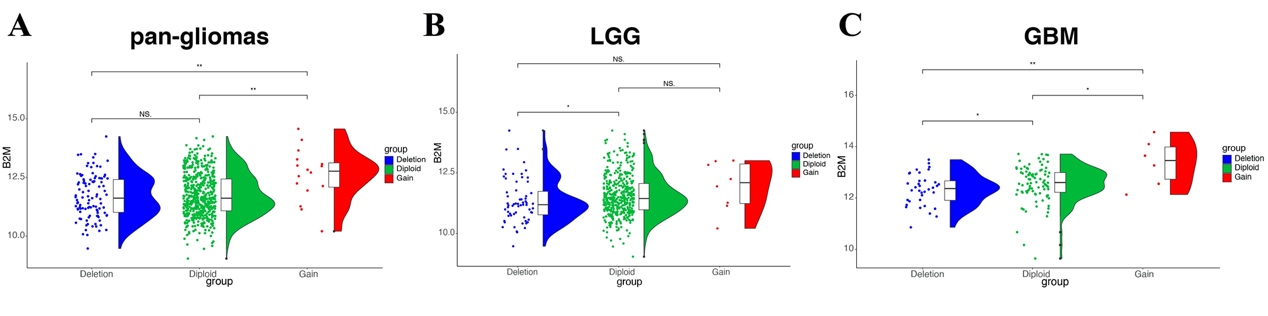


**Figure S6.** Relationship between B2M expression and copy number in **A.** TCGA pan-gliomas, **B.** TCGA LGG, and **C**. TCGA GBM.


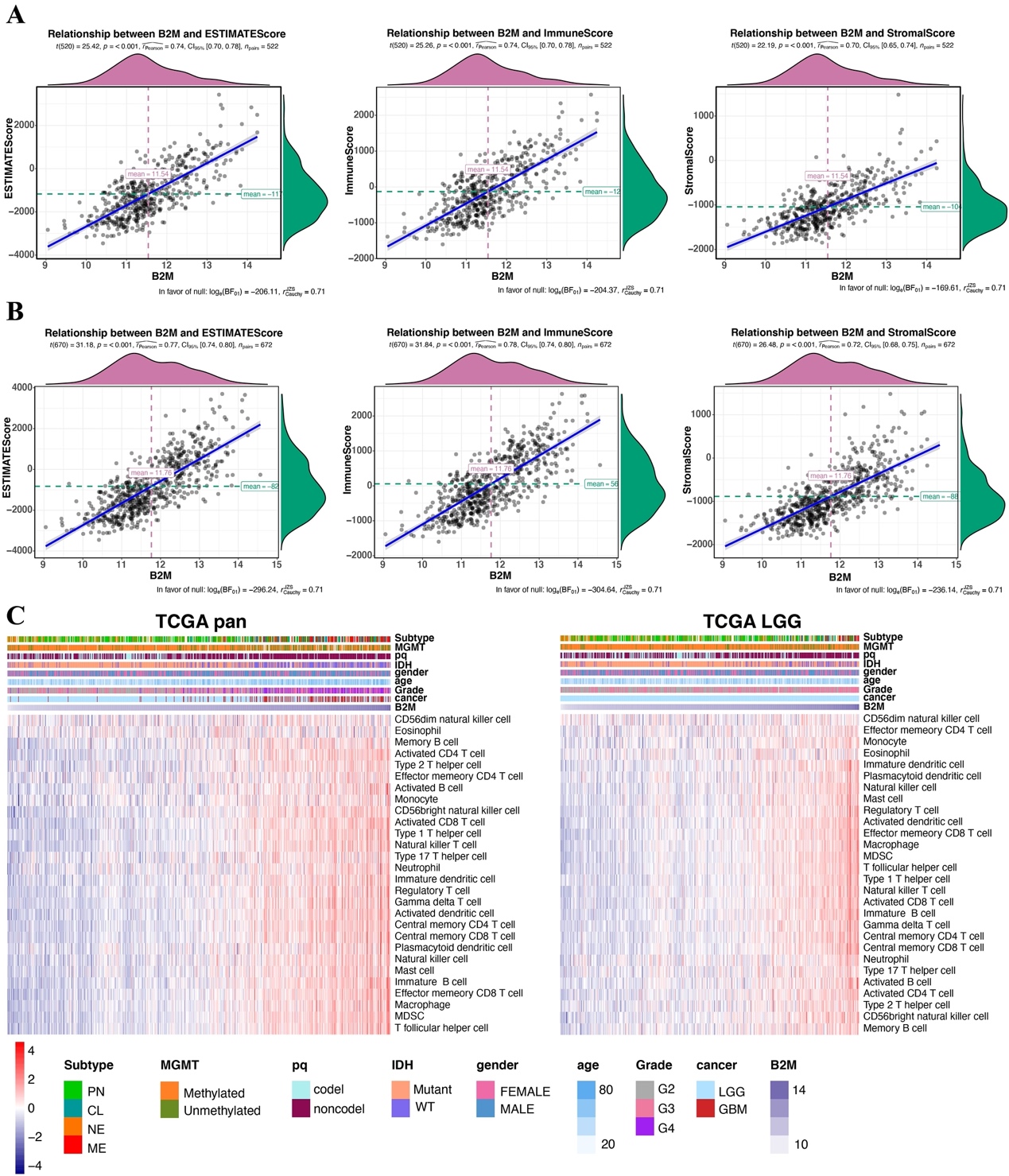


**Figure S7**. Relationship between B2M expression and ESTIMATE scores in gliomas. B2M expression was positively associated with immune score, stromal score and ESTIMATE score in LGGs (**A**) and pan-gliomas (**B**). Heatmaps illustrate the relationship between B2M and specific infiltrating cell types in pan-gliomas (**C**) and LGGs (**D**) from TCGA.


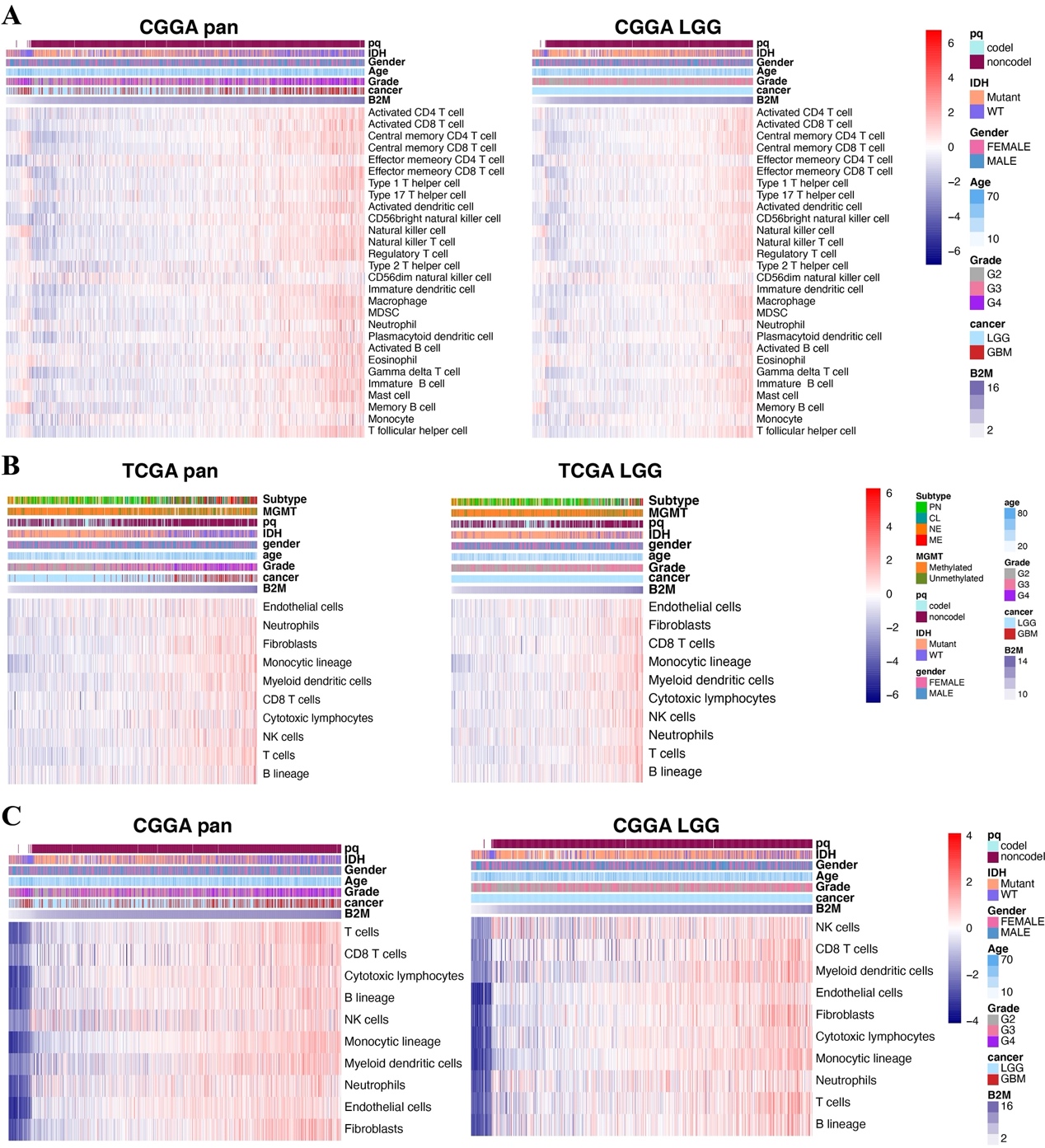


**Figure S8**. Correlation of B2M and immune cell lineages genes. **A**. B2M correlates with 28-immnue cell lineages genes in pan-gliomas and LGG in CGGA. **B**. B2M correlates with 10-immnue cell lineages genes in pan-gliomas and LGG in TCGA. **C**. B2M correlates with 10-immnue cell lineages genes in pan-gliomas and LGG in CGGA. Values are z-transformed and are highlighted in red for high expression and blue for low expression, as indicated in the scale bar.


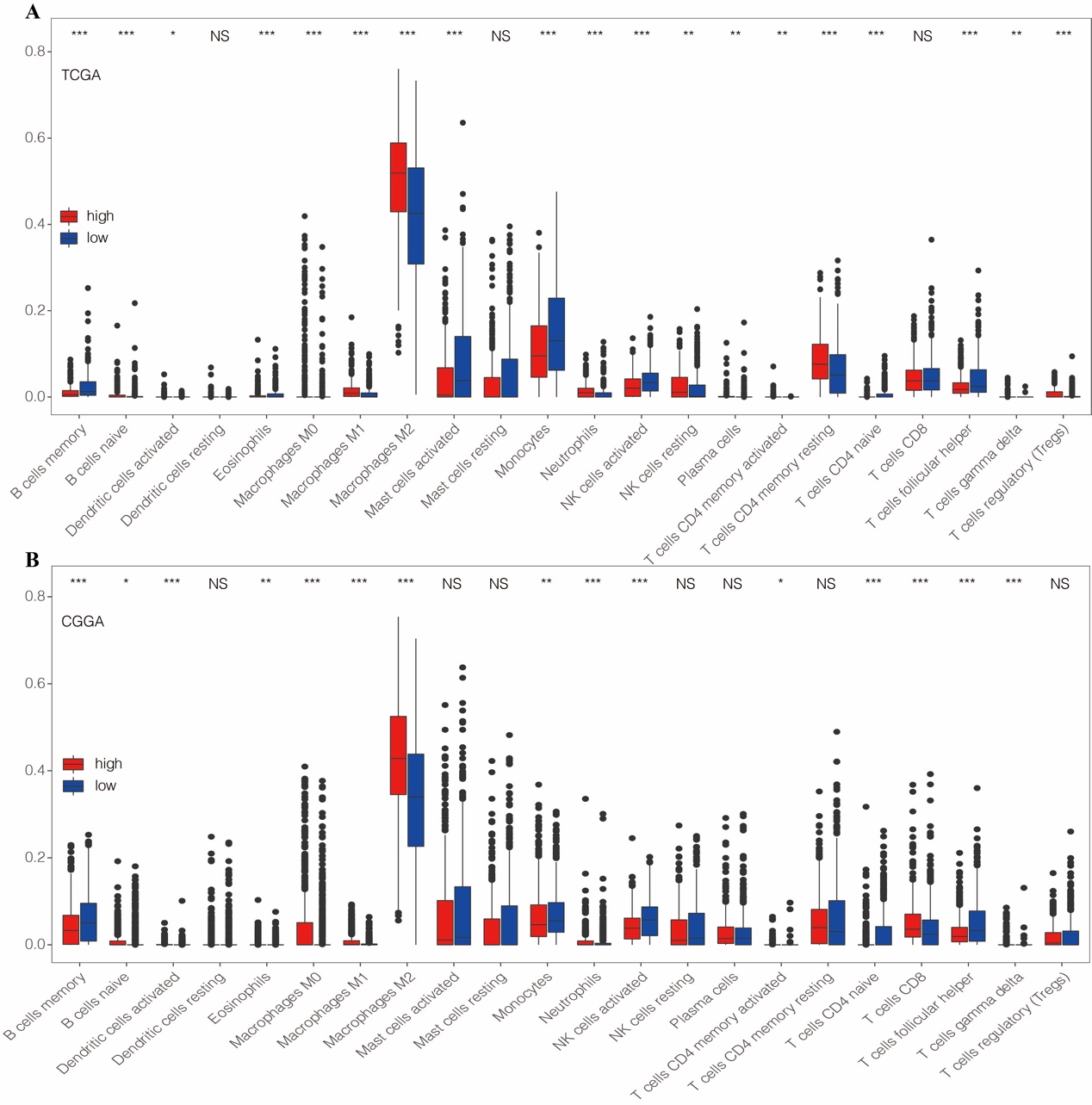


Figure S9. Box plot depicting the expression differences of B2M in the 22 immune cells types calculated by CIBERSORT algorithm in TCGA (A) and CGGA (B).


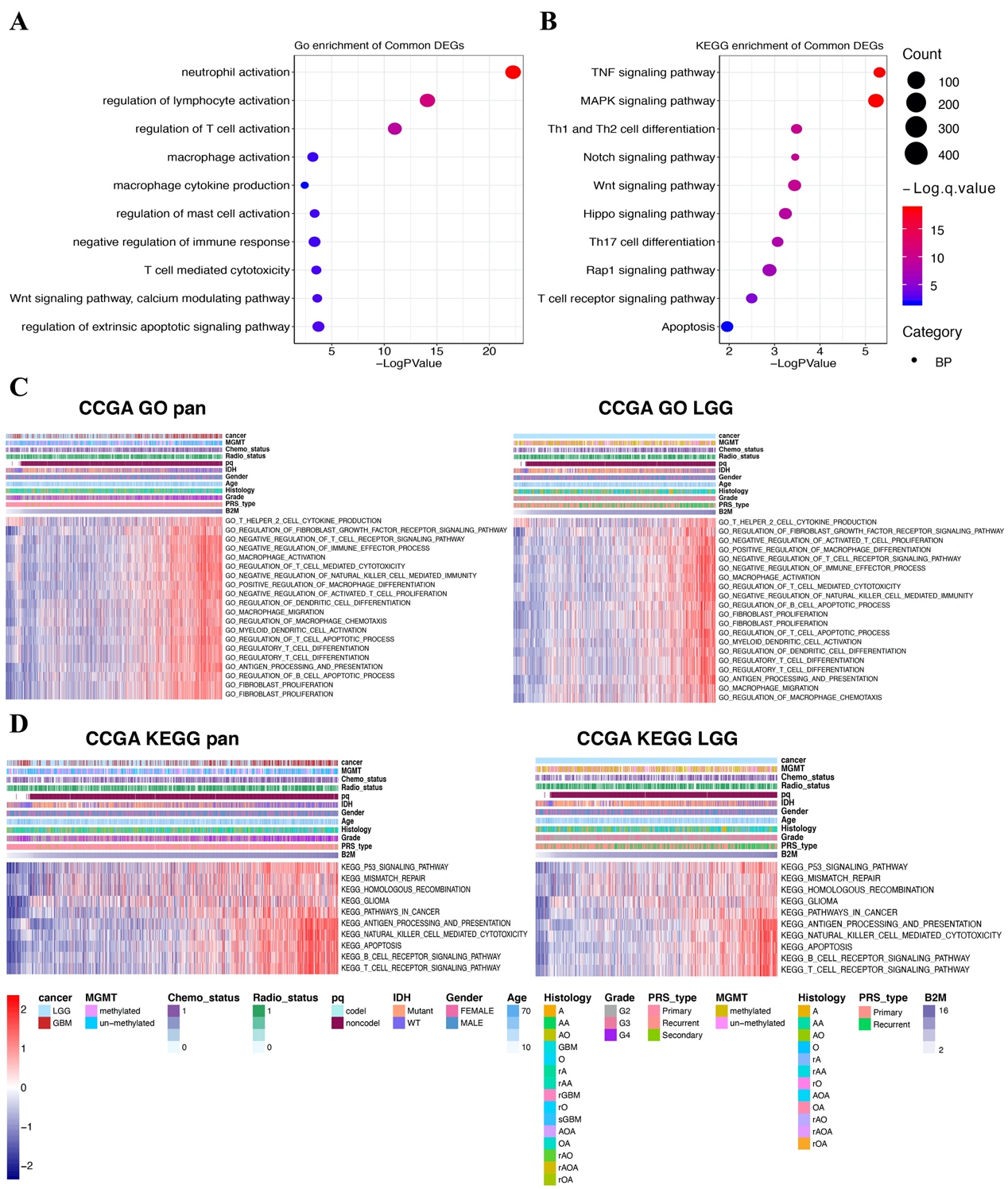


**Figure S10.** B2M-related immune functions in gliomas. **A, B.** Biological processes assess using the set of B2M-associated genes in TCGA datasets. Results are based on the GO (**A**) and KEGG (**B**) databases, respectively. **B, C.** The relationship between B2M and biological processes in pan-gliomas and LGGs from CGGA dataset. Results are based on the GO (**B**) and KEGG (**C**) databases, respectively.


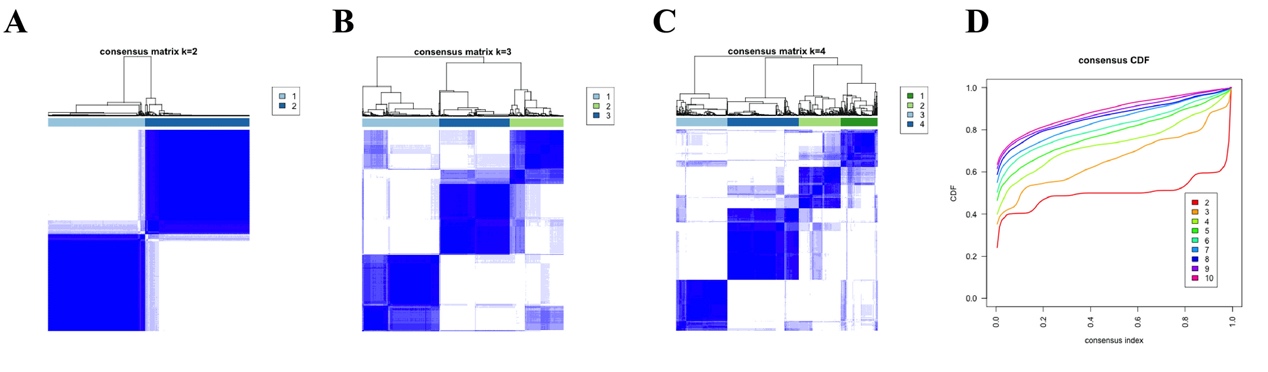


**Figure S11**. Consensus clustering of TME cell infiltration in the TCGA cohort. **A-C,** Consensus matrixes of TCGA cohorts for each k (k = 2-4), displaying the clustering stability using 1000 iterations of hierarchical clustering. **D.** Consensus clustering cumulative distribution function (CDF) for k=2 to 10 in TCGA cohorts.
